# Checkpoint-blocker-induced autoimmunity is associated with favourable outcome in metastatic melanoma and distinct T-cell expression profiles

**DOI:** 10.1038/s41416-021-01310-3

**Published:** 2021-03-15

**Authors:** Weiyu Ye, Anna Olsson-Brown, Robert A. Watson, Vincent T. F. Cheung, Robert D. Morgan, Isar Nassiri, Rosalin Cooper, Chelsea A. Taylor, Umair Akbani, Oliver Brain, Rubeta N. Matin, Nicholas Coupe, Mark R. Middleton, Mark Coles, Joseph J. Sacco, Miranda J. Payne, Benjamin P. Fairfax

**Affiliations:** 1 Oxford University Clinical Academic Graduate School, University of Oxford, Oxford, UK; 2 The Clatterbridge Cancer Centre, Wirral, UK; 3 University of Liverpool, Liverpool, UK; 4 Department of Oncology, Churchill Hospital, Oxford, UK; 5 The MRC Weatherall Institute of Molecular Medicine, University of Oxford, Oxford, UK; 6 Translational Gastroenterology Unit, John Radcliffe Hospital, University of Oxford, Oxford, UK; 7 Department of Oncology, The Christie NHS Foundation Trust, Manchester, UK; 8 Department of Dermatology, Churchill Hospital, Oxford, UK; 9 NIHR Oxford Biomedical Research Centre, Oxford University Hospitals NHS Foundation Trust, John Radcliffe Hospital, Oxford, UK; 10 Kennedy Institute of Rheumatology, NDORMS, University of Oxford, Oxford, UK

**Keywords:** Immunotherapy, Melanoma

## Abstract

**Background:**

Immune checkpoint blockers (ICBs) activate CD8^+^ T cells, eliciting both anti-cancer activity and immune-related adverse events (irAEs). The relationship of irAEs with baseline parameters and clinical outcome is unclear.

**Methods:**

Retrospective evaluation of irAEs on survival was performed across primary (*N* = 144) and secondary (*N* = 211) independent cohorts of patients with metastatic melanoma receiving single agent (pembrolizumab/nivolumab—sICB) or combination (nivolumab and ipilimumab—cICB) checkpoint blockade. RNA from pre-treatment and post-treatment CD8^+^ T cells was sequenced and differential gene expression according to irAE development assessed.

**Results:**

58.3% of patients developed early irAEs and this was associated with longer progression-free (PFS) and overall survival (OS) across both cohorts (log-rank test, OS: *P* < 0.0001). Median survival for patients without irAEs was 16.6 months (95% CI: 10.9–33.4) versus not-reached (*P* = 2.8 × 10^−6^). Pre-treatment monocyte and neutrophil counts, but not BMI, were additional predictors of clinical outcome. Differential expression of numerous gene pathway members was observed in CD8^+^ T cells according to irAE development, and patients not developing irAEs demonstrating upregulated *CXCR1* pre- and post-treatment.

**Conclusions:**

Early irAE development post-ICB is associated with favourable survival in MM. Development of irAEs is coupled to expression of numerous gene pathways, suggesting irAE development in-part reflects baseline immune activation.

## Background

Immune checkpoint blockade (ICB) therapy has transformed the outlook for metastatic melanoma (MM) patients. Current ICB standard-of-care consists of either anti-PD1 monotherapy (nivolumab or pembrolizumab, sICB), associated with a 5-year overall survival (OS) of 30–40%,^1^ or combined anti-CTLA-4/ anti-PD1 (ipilimumab and nivolumab, cICB) associated with a median OS exceeding 5 years.^2^

A key concern with ICB is the high incidence of immune-related adverse events (irAEs), especially among patients receiving cICB.^3^ IrAEs can be challenging to manage, requiring treatment interruption or discontinuation and systemic immunosuppression. Whether irAE development impacts long-term outcomes is unclear and real-world data are lacking. Several retrospective studies have observed an association between development of irAEs and improved treatment response in ICB treated MM patients, suggesting that reduced tolerance to self-antigens and reduced tolerance to tumour antigens are closely linked.^4–10^ This is not consistently observed, however,^11,12^ potentially reflecting confounding factors including differences between trial and real-world clinical populations. Within the UK National Health Service, patients are stratified to cICB or sICB depending on clinical features and patient preferences. Outside of targeted agents in the presence of an activating *BRAF* mutation, sICB recipients have access to second-line ipilimumab; whereas cICB recipients with disease progression have no further standard-of-care options.

With this in mind, we have assayed the incidence and severity of irAEs in MM patients treated with ICBs across two prospectively recruited cohorts from tertiary UK centres, to explore how early irAE development impacts clinical outcome. We have subsequently analysed CD8^+^ T-cell RNA sequencing from a subset of the cohort to investigate the relationship between gene expression and irAE development.

## Methods

### Patients

Participants were ≥18 years with diagnosed MM, receiving ≥1 cycle of ICB and provided written consent to participation (Oxford Radcliffe Biobank, 09/H0606/5 + 5, applications: OCHRe 16/A019, 18/A064). One hundred and forty-four patients were prospectively recruited between 23/11/2015–15/4/2019 (see Supplementary Data for demographics). Sixty-three patients received cICB (ipilimumab 3 mg/kg plus nivolumab 1 mg/kg 3 weekly for ≤4 treatment cycles), followed by maintenance nivolumab. Eighty-one patients receiving sICB therapy consisting of nivolumab 480 mg monthly, or pembrolizumab 2 mg/kg three weekly (69 pembrolizumab, 12 nivolumab). Seven patients had prior autoimmunity. Median number of cycles received per patient was 4 for cICB, and 8 for sICB therapy. Median follow-up duration was 18.3 (0.3–55.9) months. The replication cohort consisted of 211 patients treated at The Clatterbridge Cancer Centre, Liverpool (cICB:74, sICB:137) from 1/1/2016–7/1/2019 (HYST study: 12/NW/0525, local approval 17–18/40). Patients received ICB therapy until unacceptable irAEs, progressive disease, death or patient withdrawal. CD8^+^ T cells were isolated and RNA extracted as previously described from the Oxford cohort.^13^

### Study design

Patient demographic and clinical characteristics were collected from electronic medical records. IrAEs were reported according to the National Cancer Institute Common Terminology Criteria for Adverse Events (CTCAE) version 4.03 with pneumonitis being diagnosed via CT. Radiological response was defined by the Response Evaluation Criteria in Solid Tumours (RECIST) version 1.1,^14^ OS and PFS.

### Outcomes

We compared irAE characteristics, predictors of irAE development, and the OS and PFS in patients developing early irAEs versus those who did not. Early irAEs were defined as those before completion prior to receipt of the 5th cycle of treatment (equivalent to 12 weeks from commencement, the timepoint chosen as cICB therapy switches to maintenance nivolumab after four cycles of ipilimumab plus nivolumab). OS was defined as time from ICB starting to death from any cause. PFS was defined as the time from ICB starting to disease progression determined by serial cross-sectional imaging, or death.

### Statistical analysis

Categorical variables were summarised using frequencies and percentages, and continuous variables using medians and ranges. The time-dependent nature of developing irAEs leads to guarantee-time bias susceptibility,^15^ thus we performed a 12-week landmark analyses. Only patients alive or without disease progression at 12 weeks being included in the OS and PFS landmark analyses, respectively, patients being grouped according to irAE development prior to 12 weeks. OS and PFS was estimated using Kaplan–Meier analysis, with statistical significance determined using the log-rank test. Logistic regression was used to determine predictors of irAEs. Associations between prognostic factors and survival were investigated using univariable and multivariable Cox proportional hazards models. *P* < 0.05 was considered statistically significant and multiple testing was corrected using False Discovery Rate (FDR). Analyses were performed in R (v.3.5.1) using packages survminer,^16^ survival.^17^

### Expression analysis

Poly(A) RNA sequencing was performed as previously described,^13^ generating high-quality transcriptomes for expression analysis of pre-treatment and day 21 samples from 96 patients. Read counts were generated using HTSeq^18^ and differential expression performed using DESeq2.^19^ We controlled for the first principal component of expression, treatment, timepoint and sex. Pathway analysis was performed in XGR^20^ with the GOBP dataset,“elim” algorithm and hypergeometric test across all nominally significant (FDR < 0.5) genes. For cytokine analysis plasma samples were analysed on the Luminex cytokine platform for multiple analytes including IL-8.

## Results

### Immune-related adverse events

Treatment-related irAEs were reported in 93/144 (64.6%) patients, with 84 patients (58.3%) experiencing an irAE prior to cycle 5 and 43 (29.9%) of these patients having irAEs of grade 3/4 severity (Supplementary Table 1). There were no treatment-related deaths. Cutaneous irAEs, colitis and hepatitis occurred early post-ICB initiation (median 37, 34 and 51 days respectively); whereas gastritis and pneumonitis occurred late, with median time to onset of 386 and 378 days (Supplementary Fig. 1a, Supplementary Table 2). cICB recipients had over two-fold increase in any grade irAE frequency, and over four-fold increase in the frequency of grade 3/4 irAEs (Supplementary Data) compared to sICB recipients. Forty-one percent of cICB patients experienced irAEs affecting ≥3 organs versus 4% of patients treated with sICB (Supplementary Fig. 1b). Of those with early irAEs, 54/57 (95%) cICB and 10/27 (37%) sICB patients received steroids. ICB was temporarily interrupted in 18 (16 cICB, 2 sICB) patients and discontinued in a further 43/93 (46%) patients who experienced irAEs (31 cICB, 12 sICB). Putative risk factors for irAEs including baseline neutrophil and monocyte counts, age, sex, BMI, prior autoimmunity and treatment type were evaluated using multivariate regression. The model with the lowest Akaike Information Criteria incorporated sex, neutrophil count and treatment type, and demonstrated that only cICB treatment (OR = 27.5, 95% CI 9.8–96.5, *P* = 7.7 × 10^−9^) and neutrophil count (OR = 0.82, 95% CI 0.69–0.95, *P* = 9.7 × 10^−3^) predicted early irAEs (Supplementary Fig. 1c).

### Oncological outcomes

**Fig. 1:**
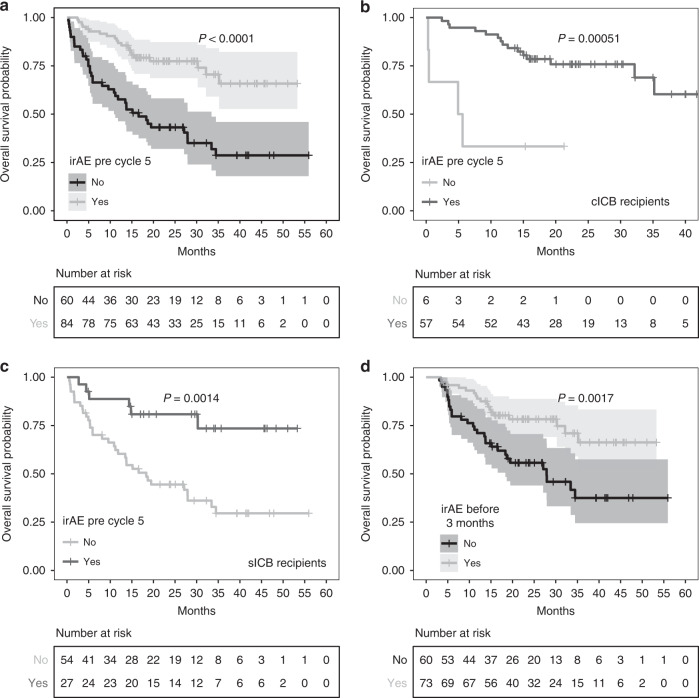
Kaplan–Meier curves of overall survival (OS) and progression-free survival (PFS) according to development of any grade of irAEs prior to cycle 5. **a** OS whole cohort, shaded areas showing 95% CI (*N* = 144), **b** Kaplan–Meier of OS specific to recipients of cICB therapy (*N* = 63), **c** Kaplan–Meier of OS specific to recipients of sICB therapy (*N* = 81), **d** 12-week landmark analysis for Oxford OS according to irAEs prior to cycle 5 (*N* = 133). All *P*-values refer to log-rank test.

Among 144 patients, 64 (44%) experienced a complete or partial response to ICB at initial radiological assessment (3-month CT), whereas 26 patients (18.1%) had stable disease, and 42 (29.2%) had progressive disease. For the remaining 11 (7.6%) patients, sequential cross-sectional imaging was unavailable; however, 9 (6.3%) patients had clear clinical progression. The median OS was 35.2 months (95% CI 30.2-Inf months), with median PFS of 18.1 months (95% CI 6.2-Inf months). The 1-year OS and PFS rates were 75% (95% CI 68–82) and 51% (95% CI 43–61), respectively. At 2 years, the OS and PFS rates were 63% (95% CI 55–72) and 40% (95% CI 31–51), respectively. Development of an early irAE prior to the 5th cycle of treatment was associated with significantly longer OS and PFS (Fig. 1a, OS *P* < 0.0001, PFS *P* = 0.00024, Supplementary Fig. 2a). This observation remained significant for OS when stratifying patients according to treatment received (Fig. 1b, c, Supplementary Fig. 2b, c). When analysis was confined to patients only developing mild grade 1/2 irAEs we again observed an OS benefit (Supplementary Fig. 2d), and this was the case when just assessing grade 3/4 irAEs (Supplementary Fig. 2e). To control for guarantee-time bias we performed a landmark analysis including only patients who are were alive (*N* = 133) or who had not progressed (*N* = 104) at 12 weeks. This again showed irAEs prior to week 12 were associated with improved OS (Fig. 1d, *P* = 0.0017), although this was not significant for PFS, indicating progression is often early (Supplementary Fig. 2f, *P* = 0.33). Finally, we performed a Cox regression model of OS, fitting irAE as a time-dependent variable, so as to incorporate data across the trajectory of follow-up, as well as age, sex and treatment type. This again demonstrated irAEs were associated with reduced hazard ratio for death (HR 0.29, 95% CI 0.15–0.58, *P* = 0.0004), whereas there were no significant associations for the other variables (*P* > 0.05).

### Independent replication

**Fig. 2:**
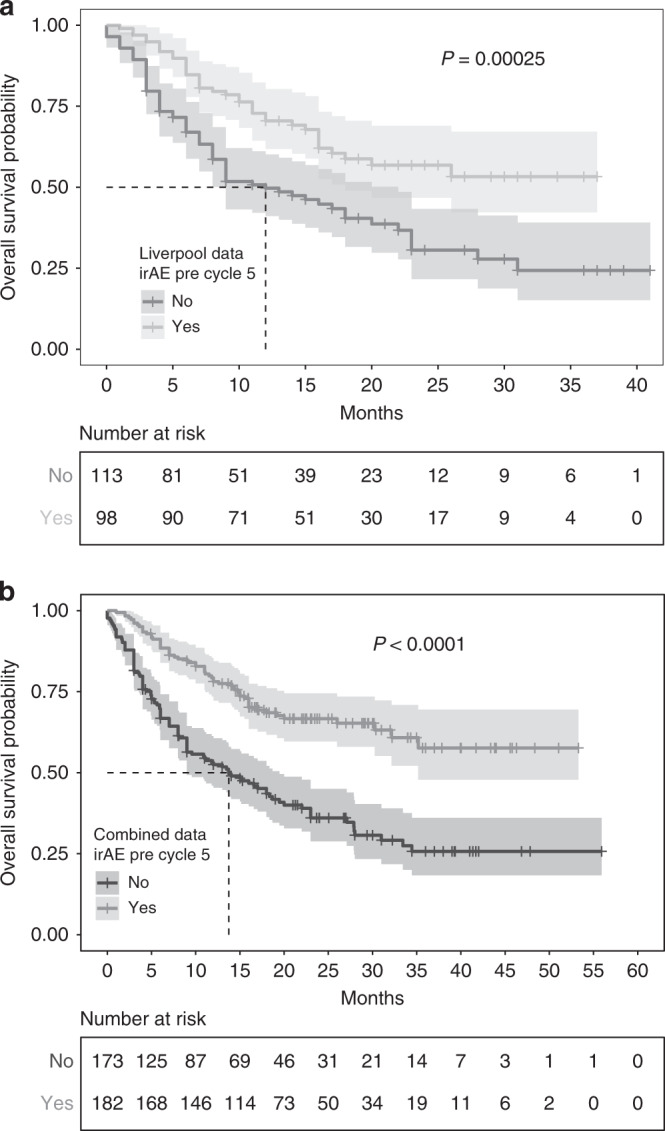
Kaplan-Meier curves of overall survival (OS) according to development of any grade of irAE prior to cycle 5 for replication cohort and combined cohort. **a** Kaplan–Meier curves of overall survival (OS) for Liverpool Replication dataset stratified according to development of irAEs prior to the fifth cycle of treatment, shaded areas showing 95% CI, (*N* = 211), **b** Kaplan–Meier curves of OS for combined Oxford and Liverpool datasets (*N* = 355). All *P*-values refer to log-rank test.

We further analysed a cohort of 211 MM patients (137 sICB, 74 cICB) receiving ICB at The Clatterbridge Cancer Centre, Liverpool with a similar demographic make-up to the Oxford cohort (Supplementary Table 3). We again found that irAEs during the first four cycles of immunotherapy were associated with longer OS (median 13 (95% CI: 8–23) months vs. not-reached, *P* = 0.00025, Fig. 2a). Assessment of each treatment type independently demonstrated a non-significant benefit of irAEs within the sICB cohort (median 15 (95% CI: 9–23) vs. 26 (95% CI: 16-Inf) months, *P* = 0.14). Conversely, this observation remained robust within the cICB cohort (median 4 (95% CI: 2-Inf) months vs not-reached (95% CI: 20-Inf), *P* = 2.2 × 10^−7^). A 12-week landmark analysis of this dataset (*n* = 183) showed that even when excluding patients who died within 3 months of treatment commencement the effect of irAE remained significant (Supplementary Fig. 3a). Combining data from both cohorts demonstrated early irAEs to be significantly associated with OS time (median 13.7 (95% CI: 9–19.5) months vs not-reached (95% CI: 35.2-Inf), *P* = 1.8 × 10^−9^, log-rank test, Fig. 2b). This remained the case for sICB alone (median 16 (95% CI: 11.8–23) months vs not-reached (95% CI: 26-Inf), *P* = 0.0017) and cICB (median 4 (95% CI: 3-Inf) months versus not-reached (95% CI: 35.2-Inf) months, *P* = 4.3 × 10^–12^). Similarly, a combined analysis of landmarked data (*n* = 316) showed a median survival for those alive after 3 months and not developing early irAE of 20 months (95% CI: 16–28 months) versus not-reached (95% CI: 35.2-Inf months, *P* = 1.1 × 10^−5^, Supplementary Fig. 3b), and this was significant for each treatment alone (sICB: *P* = 0.0054, cICB: *P* = 0.0028 cICB).

### Other variables associated with outcome

Univariable analysis demonstrated irAEs prior to cycle 5 and increased baseline albumin were associated with significantly improved OS. Conversely, non-cutaneous melanoma subtype, raised performance status, neutrophil count, monocyte count and baseline lactate dehydrogenase levels were negatively prognostic (Table 1). Retrospective analysis of MM trial data from immunotherapy and targeted agents recently indicated a protective association between BMI and clinical outcome.^21^ Interestingly, in neither the Oxford nor Liverpool cohorts did we observe an effect of BMI in either univariable or multivariable analyses of clinical outcome.

**Table 1:** Predictors of oncological outcomes using univariable and multivariable Cox proportional hazard models.

Characteristic	Univariable		Multivariable	
	HR (95% CI)	*p*-value	HR (95% CI)	*p*-value
OS Oxford				
Age (mean = 65.1)	1.01 (0.99–1.03)	0.23	—	—
Male sex	1.45 (0.86–2.45)	0.16	—	—
Performance status (>1)	2.64 (1.33–5.26)	0.0054	1.42 (0.59–3.43)	0.43
Baseline BMI	1.01 (0.97–1.05)	0.56	—	—
Baseline BMI > 25	1.36 (0.72–2.57)	0.34	—	—
irAE prior to cycle 5	0.30 (0.18–0.51)	9.8 × 10^−6^	0.28 (0.13–0.58)	0.0006^a^
Anti-PD1 treatment	1.72 (0.99–2.97)	0.054	1.23 (0.53–2.89)	0.63
Prior systemic therapy	1.60 (0.93–2.74)	0.088	1.06 (0.57–1.97)	0.85
Raised LDH (>255 iu/L)	2.28 (1.18–4.40)	0.014	—	—
Baseline neutrophil(1 × 10^6^/L)	1.21 (1.12–1.30)	5.4 × 10^−7^	1.13 (1.01–1.26)	0.029
Baseline lymphocyte(1 × 10^6^/L)	1.06 (0.71–1.56)	0.79	—	—
Baseline monocyte(1 × 10^6^/L)	12.0 (6.3–42.5)	8.3 × 10^−8^	7.00 (2.06–23.79)	0.0018^a^
Albumin	0.87 (0.82–0.93)	6 × 10^−6^	0.93 (0.87–1.01)	0.08
BRAF mutation	0.78 (0.44–1.41)	0.41	—	—
Non-cutaneous melanoma	2.8 (1.4–5.6)	0.0035	1.70 (0.73–3.93)	0.22
OS Liverpool				
Age (mean = 64.2)	0.98 (0.97–0.99)	0.005	0.98 (0.97–1.00)	0.013^a^
Male sex	1.11 (0.75–1.62)	0.61	—	—
Baseline BMI	1.01 (0.97– 1.04)	0.76	—	—
Baseline BMI > 25	1.13 (0.75–1.71)	0.56	—	—
irAE prior to cycle 5	0.48 (0.33–0.72)	0.00033	0.48 (0.33–0.72)	0.00034^a^
Anti-PD1 treatment	1.28 (0.82–1.89)	0.32	—	—
Prior systemic therapy	2.09 (1.05–4.16)	0.04	1.78 (0.87–3.56)	0.12
Baseline neutrophil (1 × 10^6^/L)	1.20 (1.10–1.31)	2.2 × 10^−5^	—	—
Baseline lymphocyte (1 × 10^6^/L)	0.80 (0.53–1.21)	0.29	—	—
Low albumin (<36 g/dl)	7.33 (3.50–15.35)	1.3 × 10^−7^	—	—

Multivariate analysis only performed on variables where 95% or more of values were available for analysis. Albumin threshold as determined for normal range by institution.

^a^Significant after FDR correction for multiple testing.

**Fig. 3:**
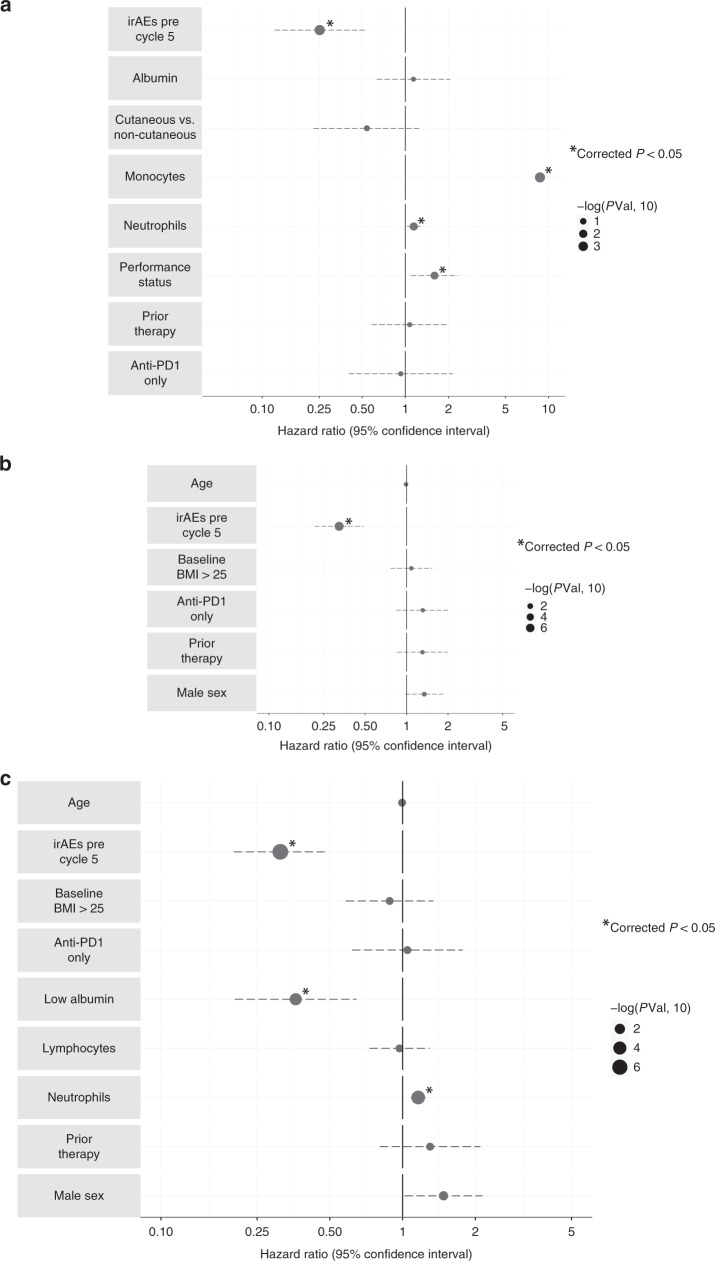
Factors associated with overall survival (OS). **a** Results from multivariable Cox Proportional Hazard analysis of factors associated with overall survival (OS) in Oxford cohort (*N* = 138), *P*-values corrected for multiple testing cohort, **b** Factors associated with OS across combined Oxford and Liverpool datasets where data are available for all individuals (*N* = 350), **c** Factors associated with OS across combined Oxford and Liverpool datasets where data available include cell counts (*N* = 276).

Multivariable analysis demonstrated early irAEs, monocyte count and neutrophil count were nominally associated with OS (Table 1), with irAEs and monocyte count remaining significant after correcting for multiple testing (Table 1, Fig. 3a). In univariable analyses of the Liverpool cohort, low albumin, increased neutrophil count and prior systemic treatment were negatively prognostic; whereas early irAEs and increasing age were protective (Table 1).

We combined the datasets to provide increased power in multivariable analyses of prognostic factors across institutions (*N* = 350). Across both datasets early irAEs were associated with a 0.32 hazard ratio (95% CI: 0.22–0.49, *P* = 6.7 × 10^−8^, Table 2, Fig. 3b). Multivariable analysis of 276 individuals with cell counts demonstrated increased baseline neutrophil count (HR 1.16 per unit, 95% CI: 1.08–1.24, *P* = 2.8 × 10^−5^) and low albumin (HR 2.77, 95% CI: 1.55–4.95, *P* = 5.8 × 10^−4^) were negatively associated with oncological outcome (Table 2, Fig. 3c).

**Table 2:** Univariable and multivariable analyses of factors associated with OS—combined datasets.

Characteristic	Univariable		Multivariable	
	HR (95% CI)	*p*-value	HR (95% CI)	*p*-value
OS—baseline indices (*n* = 348)				
Age	0.99 (0.98–1.00)	0.24	0.99 (0.98–1.00)	0.15
Male sex	1.24 (0.91–1.68)	0.18	1.34 (0.98–1.85)	0.07
Baseline BMI	1.01 (0.98–1.03)	0.63	—	—
Baseline BMI > 25	1.13 (0.81–1.60)	0.46	1.08 (0.77–1.53)	0.65
irAE prior to cycle 5	0.39 (0.28–0.54)	6.4 × 10^−9^	0.32 (0.22–0.49)	6.7 × 10^−8^
Single agent anti-PD1	1.48 (1.06–2.06)	0.02	0.76 (0.49–1.19)	0.23
Prior systemic therapy	1.36 (0.91–2.03)	0.14	1.31 (0.86–1.99)	0.21
OS—including biochemical and haematological indices (*n* = 276)				
Age	—	—	1.01 (0.98–1.01)	0.53
Male sex	—	—	1.48 (1.02–2.14)	0.04
Baseline BMI > 25	—	—	0.88 (0.58–1.34)	0.56
irAE prior to cycle 5	—	—	0.31 (0.20–0.49)	2.5 × 10^−7^
Anti-PD1 treatment	—	—	1.05 (0.62–1.77)	0.86
Prior systemic therapy	—	—	1.30 (0.8–2.1)	0.28
Baseline neutrophil	1.19 (1.13–1.26)	4.9 × 10^−10^	1.16 (1.08–1.24)	2.8 × 10^−5^
Baseline lymphocyte	1.11 (0.66–1.23)	0.53	0.97 (0.73–1.29)	0.84
Low albumin (per centre)	4.7 (2.87–7.70)	8.1 × 10^−10^	2.77 (1.55–4.95)	5.8 × 10^−4^

Multivariate analysis only performed on variables where 95% or more of values were available for analysis. Albumin threshold as determined for normal range by institution.

### Association of irAE development with divergent CD8^+^ T-cell gene expression

**Fig. 4:**
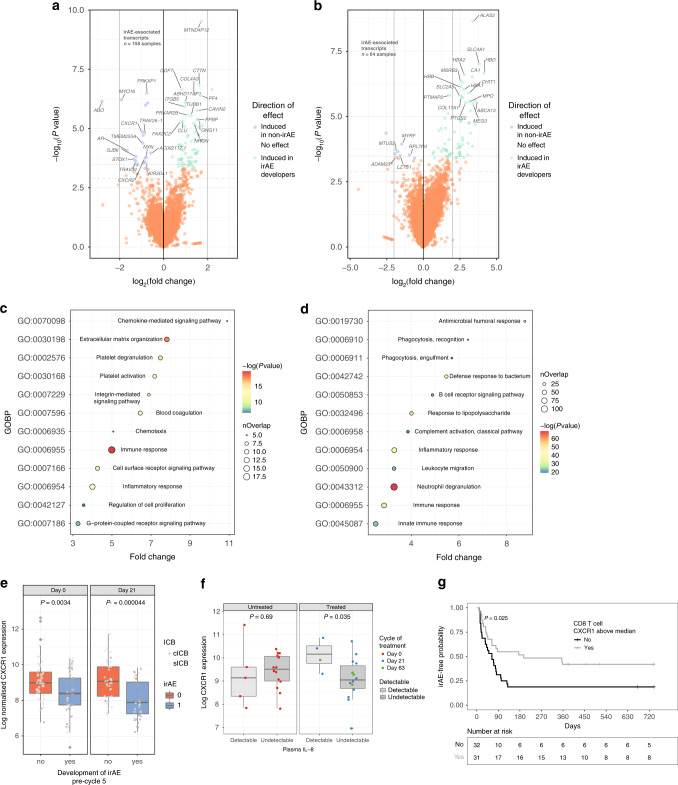
Differential gene expression in CD8+ T cells according to development of irAEs. **a** Volcano plot of differentially expressed transcripts from 79 patients (158 samples) taken pre- and post-treatment, controlling for cycle and treatment type. Each point represents a transcript, with those in blue having increased expression in those not developing irAE, whereas those in green are raised in those developing irAE; **b** As per **a** but samples were confined to pre-treatment from sICB recipients; **c**, **d** Go Ontology Biological Process (GOBP) pathway analysis of genes nominally associated (FDR < 0.5) with irAE development from **a** and **b**, respectively (*y*-axis: GOBP code, *x*-axis: fold change enrichment); **e** Boxplots demonstrating association of *CXCR1* expression with irAE development from pre- and post-treatment samples; **f** *CXCR1* expression according to detectable plasma IL-8 cytokine measurements from samples pre (left panel) and post ICB; **g** Kaplan–Meier curve of time to irAE development in patients who survived for >1-year post-treatment according divided along the lines of median CD8^+^ T-cell *CXCR1* expression at day 21.

Identification of markers predictive of irAEs is of high interest to immuno-oncology^22^ and peripheral CD8^+^ T-cell expression profiles are associated with clinical outcome.^13^ We therefore investigated the relationship between irAEs and peripheral CD8^+^ T-cell expression from pre- (day 0) and post-treatment (day 21) samples from patients in the Oxford cohort, correcting for treatment type, cycle of treatment, and the first principal component (*N* = 79 patients, 158 samples). In this analysis we found 50 transcripts were differentially associated with development of early irAEs (FDR < 0.05, Fig. 4a, Supplementary Table 4). When analysis was confined to the pre-treatment samples, we did not see an association of CD8^+^ T-cell expression with irAE development in those who received cICB, whereas in patients who received sICB (*N* = 64) we found irAE development was associated with differential expression of 35 transcripts (FDR < 0.05, Fig. 4b, Supplementary Table 4). Pathway analysis of transcripts nominally associated with irAE development across cycles of treatment (FDR < 0.5) showed gene pathways including chemokine-mediated signalling, extracellular matrix organisation and platelet degranulation to be altered in irAE development (Fig. 4c, Supplementary Table 5), while analysis of those associated with subsequent development of irAEs in sICB recipients including pathways involved in antimicrobial responses, phagocytosis and complement activation (Fig. 4d, Supplementary Table 5).

### *CXCR1* and irAE

We found increased expression of *CXCR1*, and to a lesser extent *CXCR2* (Supplementary Table 4), encoding chemokine receptors for IL-8, to be associated with reduced development of irAEs pre- and post-treatment (Fig. 4e). Given circulating IL-8 levels are negatively prognostic across ICB treatments^23^ we explored the relationship between plasma IL-8 and CD8^+^ *CXCR1* expression (*N* = 19 patient samples). Notably we found post-treatment samples from individuals with detectable plasma IL-8 (>1 pg/ml) showed increased *CXCR1* expression, in keeping with raised levels of IL-8 potentially directly influencing CD8^+^ subsets (Fig. 4f). We further explored the relationship between *CXCR1* expression and irAE development, restricting analysis to patients alive beyond 1-year post-treatment commencement (*N* = 63 samples with expression data). Dividing the cohort according to median day 21 *CXCR1* expression we noted a significant association between those with reduced *CXCR1* expression and time to develop an irAE (*P* = 0.025, log-rank test, Fig. 4g), further supporting a key role for this pathway in ICB response.

## Discussion

Consistent with the literature,^3^ irAEs occur at a markedly higher incidence in patients treated with cICB compared to sICB and are typically more severe and more likely to involve multiple organs. We also find increased neutrophil count was associated with reduced irAE incidence. In this study neither sex nor autoimmune history influenced the development of early irAEs.^24^ The lack of sex effect suggests classical risk factors for autoimmune disease may be less relevant to ICB-associated irAEs. In keeping with others, we find distinct irAEs have a dissimilar median time-to-onset, likely reflecting divergent pathophysiology. Cutaneous irAEs, colitis and hepatitis typically occur early, while late complications include gastritis and pneumonitis. The timescales within our cohort broadly corroborate those reported in previous studies,^4,8,11,25^ except for pneumonitis which occurred late at a median time of 378 days.

Associations between irAEs and treatment response in MM are limited to trials,^4,5,7^ with vitiligo, long-known to have favourable associations with melanoma outcome, linked to an objective response to ICB therapy.^9,10^ A link between irAEs and survival benefit in ICB therapy has also been reported in non-small cell lung cancer.^26^ However, not all evidence supports a link between irAEs and improved survival.^8,11,12^ Here we demonstrate in the standard-of-care setting that development of irAEs of any grade is associated with improved OS. This observation remains significant in the 12-week landmark analysis, ruling out a guarantee-time bias where patients died before being able to develop irAEs. Importantly, we replicated these observations in an independent cohort, with treatment-specific analysis showing the effect being highly significant in recipients of cICB, and a non-significant directional trend in the sICB group. We note irAEs are not an absolute requirement for oncological response, and many patients (32% of those alive across both cohorts at 1 year) showed response to treatment in the absence of early irAE. Multivariable analyses of the combined datasets, however, demonstrated irAE development was associated with outcome across treatments. Similarly, raised pre-treatment performance status, neutrophil and monocyte counts were negatively prognostic.

Given irAEs were frequently managed with steroids and other immunosuppressants, these treatments are unlikely to adversely affect prognosis. Conversely, our results suggest separating efficacy from irAE propensity in novel agents may be difficult. Finally, in contrast to recent cross-treatment analysis, we did not observe an association between BMI and clinical outcome^21^ in either the Oxford or Liverpool cohorts in either sex. Importantly BMI was collected prospectively, and analysis was restricted to immunotherapy recipients. These results suggest any link between BMI and favourable outcome in ICB is not clear-cut and dedicated prospective studies may add clarity.

Analysis of CD8^+^ T-cell gene expression and irAE development identified divergent gene expression according to irAE development across pre-and post-treatment samples. We could additionally identify baseline changes in sICB recipients who proceeded to develop irAE with enrichment in complement activity, innate immunity and neutrophil degranulation pathways. The presence of these innate immune signatures in CD8^+^ T cells may reflect increased inter-cellular adherence, a common finding in activated T cells.^27^ Given cICB elicits markedly greater CD8^+^ expression changes than sICB,^13^ we postulate baseline variation in expression is less important compared to the effect of treatment. Thus, our data suggest propensity to develop irAEs post-sICB is in part due to baseline CD8^+^ T-cell activation, which may reflect pre-treatment anti-tumour responses. Common to baseline and treated samples was the observed differential expression of *CXCR1*, with raised expression in those not developing irAEs. Notably, plasma IL-8, a key cytokine mediator of neutrophil chemotaxis and ligand of CXCR1, is strongly associated with clinical outcomes to ICB treatment.^23^ In a subset of samples, we measured IL-8 levels, finding those with detectable plasma IL-8 post-treatment show increased expression of T-cell *CXCR1*, linking levels of receptor and ligand. Further, we observe that day 21 *CXCR1* is associated with time to develop an irAE. These observations provide further weight to evidence linking tumour-independent immune parameters with clinical outcome to ICB.

Our study reflects real-world scenarios, with particular relevance to the UK healthcare setting, and our observations independently replicate in a separate tertiary centre. Our ability to combine clinical observations with prospectively collected transcriptomic data identified divergences in peripheral CD8^+^ T-cell gene expression linked to irAE development and implicate the IL-8: CXCR1 axis in this process. Limitations include the retrospective collection of clinical data and the relatively small sample size in the primary cohort. Larger transcriptomic series involving other cell subsets will be vital in understanding the relationship between clinical response and irAE development. A positive association between increased tumour mutational burden (TMB) and response to ICB is well recognised,^28^ and we speculate that TMB may relate to irAE development, with the increased neo-antigen burden of high TMB leading to off target effects. The clinical utility of these findings will require prospective trials, but our results suggest that patients with raised neutrophil counts have reduced risk of irAE development and poorer prognosis—arguing for treatment with cICB. Conversely, in patients without oncological responses to sICB who have not developed irAE, there might be argument that effective immune stimulation has not been elicited and switching to cICB could be helpful.

In conclusion, in our clinical practice early irAE development post-ICBs is associated with a strong survival benefit in MM that robustly reproduces in an independent centre. Furthermore, irAE development is associated with divergent patterns of peripheral CD8^+^ T-cell gene expression, with raised expression of *CXCR1* being associated with reduced irAE development. Collectively, these observations significantly further our insights into the clinical and immunological significance of irAEs and may assist in the application of stratified medicine.

## Supplementary information

Supplementary Figures & Tables

## Data Availability

Sequencing data deposited at European Genome–phenome Archive under accession no. EGAS00001004081, available via a data access agreement.

## Supplementary information

**Supplementary information** The online version contains supplementary material available at 10.1038/s41416-021-01310-3.
