# Supplementary material for: Checkpoint-blocker-induced autoimmunity is associated with favourable outcome in metastatic melanoma and distinct T-cell expression profiles
Source: Br J Cancer. 2021 Mar 15;124(10):1661–9. doi: 10.1038/s41416-021-01310-3 (PMC8110747; doi:10.1038/s41416-021-01310-3)
Supplement: Supplementary file 1 — Supplementary Figures & Tables [file 41416_2021_1310_MOESM1_ESM.pdf]

Supplementary Information

Checkpoint-blocker induced autoimmunity is associated with favourable outcome in metastatic melanoma and distinct T cell expression profiles

Weiyu Ye, Anna Olsson-Brown, Robert A. Watson, Vincent T. F. Cheung, Robert D. Morgan, Isar Nassiri, Rosalin Cooper, Chelsea A. Taylor, Umair Akbani, Oliver Brain, Rubeta N. Matin, Nicholas Coupe, Mark R. Middleton, Mark Coles, Joseph J. Sacco, Miranda J. Payne, Benjamin P. Fairfax

Supplementary Figure 1. Profile of immune-related adverse events (irAEs) within the cohort. a) Density plot of the development of different irAEs over time. b) Distribution of the number of different irAEs per patient. c) Logistic regression model of the risk factors for development of irAEs prior to cycle 5

Supplementary Figure 2. a) Kaplan Meier of Oxford progression free survival (PFS) for all recipients, b) Kaplan Meier of Oxford PFS for cICB recipients, c) Kaplan Meier of Oxford PFS for sICB recipients, d) Kaplan Meier of Oxford overall survival (OS) based on development of grade 1/grade 2 or e) grade 3/grade 4 toxicity prior to cycle 5, f) Landmark analysis for Oxford PFS according to irAEs prior to cycle 5, (all P values from log-rank test, shaded areas showing 95% CI).

Supplementary Figure 3. a) 12 week landmark analysis for Liverpool OS according to irAEs prior to cycle 5 (*N*=183). b) 12 week landmark analysis for combined datasets according to irAEs prior to cycle 5 (*N*=316).

Supplementary Data

Supplementary Table 1. Baseline demographic and clinical characteristics of patients

Supplementary Table 2. Immune-related adverse events (irAEs) across the Oxford cohort

Supplementary Table 3. Comparison of Oxford and Liverpool cohorts

Supplementary Table 4. List of genes differentially associated with irAE development

Supplementary Table 5. List of GOBP pathways enriched for members of genes associated with irAE development

Supplementary Figure 1

a

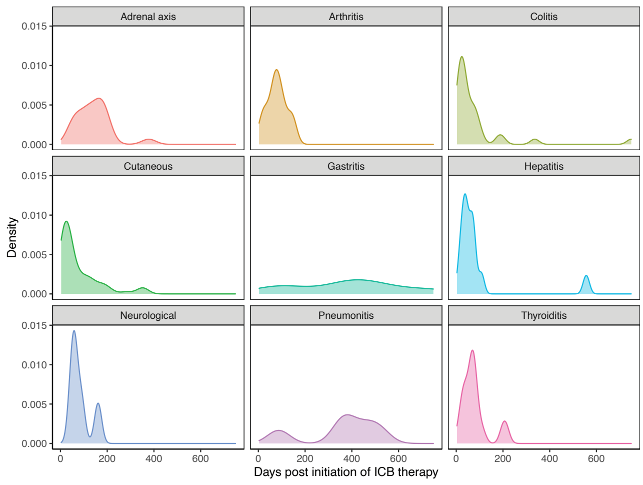

b

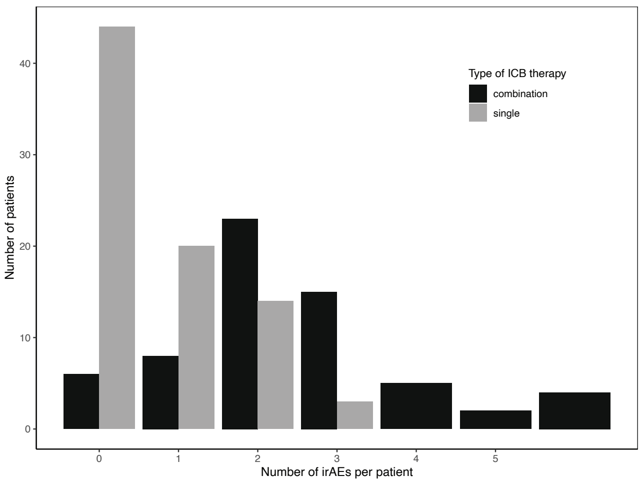

c

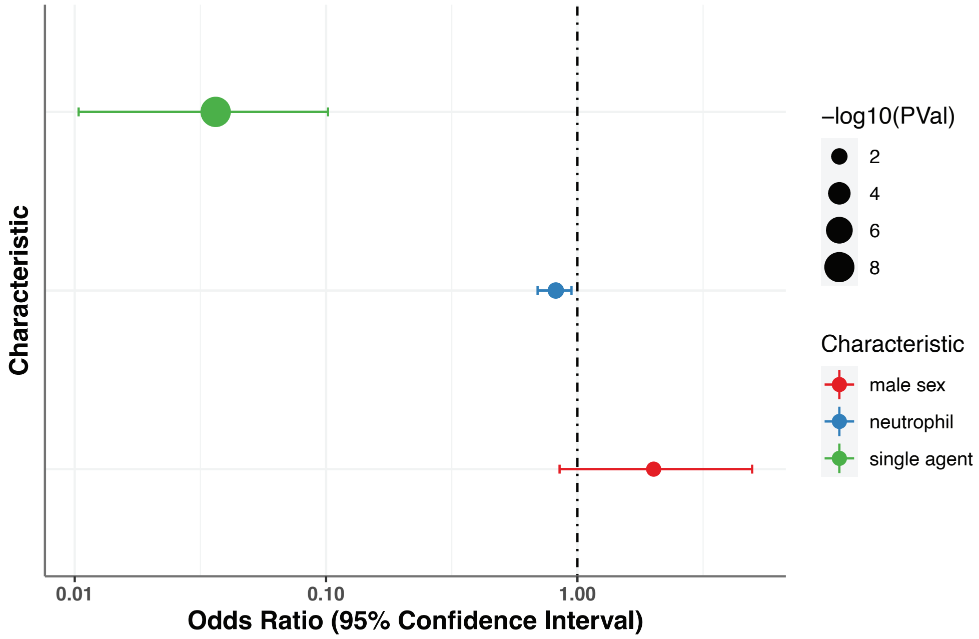

Supplementary Figure 2

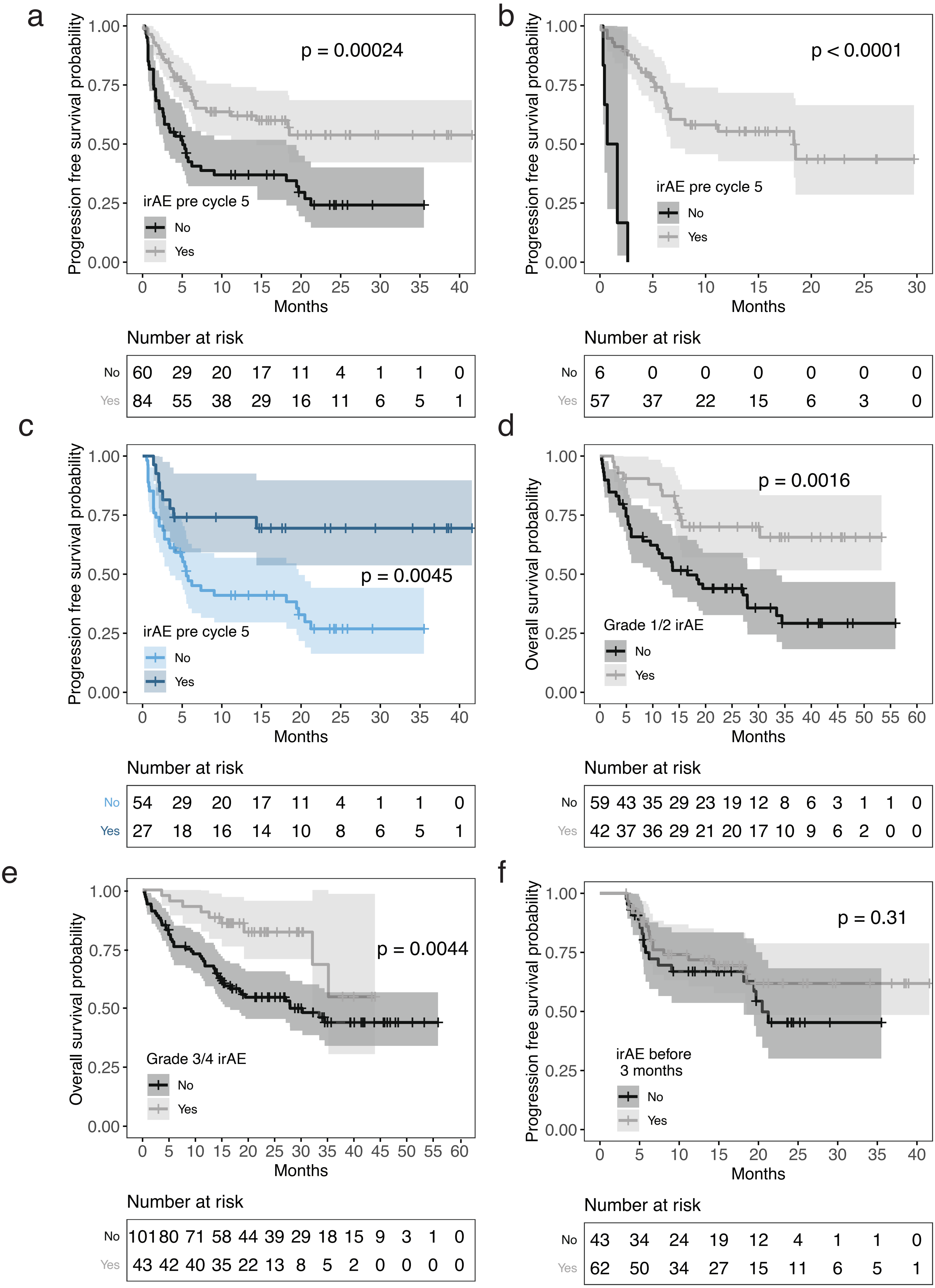

a — Number at risk

No	60	29	20	17	11	4	1	1	0
Yes	84	55	38	29	16	11	6	5	1

b — Number at risk

No	6	0	0	0	0	0	0
Yes	57	37	22	15	6	3	0

c — Number at risk

No	54	29	20	17	11	4	1	1	0
Yes	27	18	16	14	10	8	6	5	1

d — Number at risk

No	59	43	35	29	23	19	12	8	6	3	1	1	0
Yes	42	37	36	29	21	20	17	10	9	6	2	0	0

e — Number at risk

No	101	80	71	58	44	39	29	18	15	9	3	1	0
Yes	43	42	40	35	22	13	8	5	2	0	0	0	0

f — Number at risk

No	43	34	24	19	12	4	1	1	0
Yes	62	50	34	27	15	11	6	5	1

Supplementary Figure 3

a

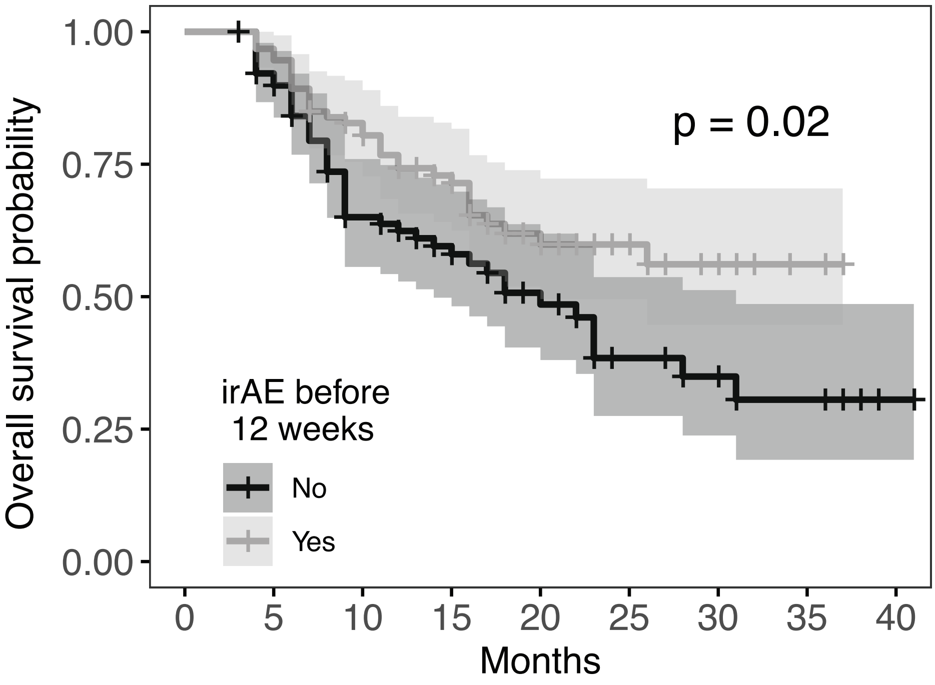

Number at risk

No	90	81	51	39	23	12	9	6	1
Yes	93	90	71	51	30	17	9	4	0

b

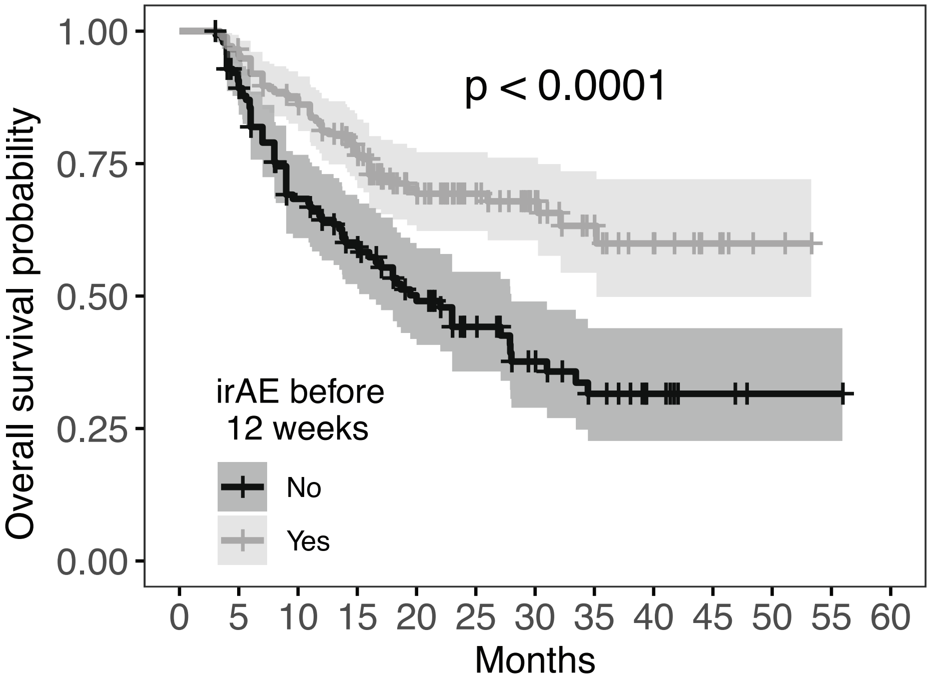

Number at risk

No	141	125	87	69	46	31	21	14	7	3	1	1	0
Yes	175	168	146	114	73	50	34	19	11	6	2	0	0

Table 1. Baseline demographic and clinical characteristics of patients

Characteristic	Single ICB therapy (n=81)	Combination ICB therapy (n=63)	Complete cohort (n=144)
Median age (range), years	74 (29 – 95)	59 (21 – 74)	68 (21 – 95)
Males, n (%)	44 (55.4)	35 (55.6)	79 (54.9)
Median BMI (range)	28 (20 – 59)	28 (19 – 43)	28 (19 – 59)
Prior autoimmune disease, n (%)	4 (4.9)	3 (4.7)	7 (4.9)
ECOG performance status, n (%)			
0	31 (38.3)	48 (76.2)	79 (54.9)
1	35 (43.2)	12 (19.0)	47 (32.6)
2	12 (14.8)	3 (4.8)	15 (10.4)
3	2 (2.5)	0 (0.0)	2 (1.4)
Not reported	1 (1.2)	0 (0.0)	1 (0.7)
BRAF status, n (%)			
Mutation	19 (23.4)	24 (38.1)	43 (29.9)
Wild type	54 (66.7)	31 (49.2)	85 (59.0)
Not reported	8 (9.9)	8 (12.7)	16 (11.1)
Elevated LDH, n (%)	23 (27.7)	20 (31.7)	43 (29.9)
Prior systemic treatment, n (%)	27 (33.3)	9 (14.3)	36 (25)
BRAF inhibitor	10 (12.3	6 (9.5)	16 (11.1)
Ipilimumab	17 (21.8)	1 (1.6)	18 (12.3)
Other	0 (0.0)	3 (4.7)	3 (2.2)

Supplementary Table 2. Immune related adverse events (irAEs) across the cohort

irAE	Median time to irAE onset (days)	Single ICB therapy (n=81)		Combination ICB therapy (n=63)	
		Any grade irAE, n (%)	Grade 3/4 irAE, n (%)	Any grade irAE, n (%)	Grade 3/4 irAE, n (%)
Total	-	36 (44.4)	11 (13.6)	57 (90.4)	39 (61.9)
Cutaneous manifestation	37	27 (33.3)	0 (0.0)	34 (55.0)	3 (4.8)
Colitis	34	5 (6.2)	1 (1.2)	27 (42.9)	13 (20.6)
Hepatitis	51	2 (2.5)	1 (1.2)	23 (36.5)	14 (22.2)
Adrenal axis*	132	5 (6.2)	5 (6.2)	16 (25.4)	14 (22.2)
Thyroiditis	68	2 (2.6)	0 (0.0)	14 (22.2)	1 (1.6)
Arthritis	79	5 (6.2)	1 (1.2)	7 (11.1)	1 (1.6)
Pneumonitis	378	3 (3.7)	1 (1.2)	2 (3.2)	2 (3.2)
Gastritis	386	3 (3.7)	2 (2.5)	4 (6.3)	2 (3.2)
Neurological	63	0 (0.0)	0 (0.0)	5 (7.9)	2 (3. 2)
Nephritis	126	2 (2.5)	1 (1.2)	3 (4.8)	1 (1.6)
Diabetes	65	0 (0.0)	0 (0.0)	2 (3.2)	2 (3.2)
Myositis	49	1 (1.2)	0 (0.0)	2 (3.2)	2 (3.2)
Haematological	206	0 (0.0)	0 (0.0)	2 (3.2)	1 (1.6)

*adrenal axis encompasses hypophysitis and adrenal insufficiency

Supplementary Table 3. Oxford vs. Liverpool characteristics

Characteristic	sICB (n=136)	P vs. Oxford	cICB (n=74)	P vs. Oxford	All treatments		
					Liverpool cohort (n=210)	Oxford cohort (n=144)	P
Median age (range), years	68 (28 – 89)	P=0.002 (KS test)	65 (29 – 87)	P=0.048 (KS test)	66 (28 – 89)	68 (21-95	0.51
Males, n (%)	77 (56.6)	P=0.80	42 (56.7)	P=1	119 (56.7)	79 (54.9)	0.74
Median BMI (range)	28 (21 – 59)	P=0.24 (KS test)	27 (19 – 43)	P=0.44 (KS test)	27 (17-49)	28 (19 – 59)	0.12

Table 4: Differential expression results

baseMean	log2FoldChange	lfcSE	stat	pvalue	padj	ENSG.gene	Gene	dataset
51.54071	1.7102988	0.2714468	6.300678	0.0000000	0.0000052	ENSG00000247627	MTND4P12	pre/post treatments
47.57524	1.6157868	0.3075940	5.252985	0.0000001	0.0009941	ENSG00000085733	CTTN	pre/post treatments
28.90689	2.1865431	0.4223234	5.177414	0.0000002	0.0009941	ENSG00000236304	LOC107984360	pre/post treatments
34.09527	0.8382315	0.1619171	5.176917	0.0000002	0.0009941	ENSG00000143869	GDF7	pre/post treatments
220.78798	-0.7597212	0.1485712	-5.113516	0.0000003	0.0010148	ENSG00000259205	PRKXP1	pre/post treatments
28.58393	1.4357006	0.2816862	5.096808	0.0000003	0.0010148	ENSG00000169031	COL4A3	pre/post treatments
165.34309	1.6872416	0.3331864	5.063957	0.0000004	0.0010341	ENSG00000163737	PF4	pre/post treatments
19.03734	-1.9123003	0.3836662	-4.984281	0.0000006	0.0013343	ENSG00000041515	MYO16	pre/post treatments
31.81067	-2.7948843	0.5638178	-4.957070	0.0000007	0.0013343	ENSG00000175164	ABO	pre/post treatments
33.38386	0.9864350	0.1999462	4.933501	0.0000008	0.0013343	ENSG00000198658	ABHD17AP1	pre/post treatments
567.48983	-0.7212410	0.1463690	-4.927552	0.0000008	0.0013343	ENSG00000270127	AC027020.2	pre/post treatments
47.72806	-0.8192674	0.1677174	-4.884808	0.0000010	0.0015208	ENSG00000251819	RNU6-322P	pre/post treatments
62.44311	0.9633340	0.1982718	4.858653	0.0000012	0.0016025	ENSG00000082781	ITGB5	pre/post treatments
166.32468	1.5288752	0.3196692	4.782679	0.0000017	0.0021779	ENSG00000168497	CAVIN2	pre/post treatments
200.63978	1.0835715	0.2307193	4.696493	0.0000026	0.0031102	ENSG00000005249	PRKAR2B	pre/post treatments
379.57833	1.2527232	0.2676210	4.680959	0.0000029	0.0031457	ENSG00000101162	TUBB1	pre/post treatments
341.15760	1.5442639	0.3353949	4.604316	0.0000041	0.0041301	ENSG00000163736	PPBP	pre/post treatments
64.01254	1.5564446	0.3383306	4.600365	0.0000042	0.0041301	ENSG00000127920	GNG11	pre/post treatments
554.12112	0.8759816	0.1918116	4.566885	0.0000050	0.0045669	ENSG00000120885	CLU	pre/post treatments
99.09008	0.7950417	0.1744547	4.557295	0.0000052	0.0045669	ENSG00000170271	FAXDC2	pre/post treatments

Table 4: Supplementary Table 4 *(continued)*

baseMean	log2FoldChange	lfcSE	stat	pvalue	padj	ENSG.gene	Gene	dataset
402.03183	1.3738071	0.3036746	4.523945	0.0000061	0.0050948	ENSG00000154146	NRGN	pre/post treatments
261.15059	1.4476915	0.3231597	4.479802	0.0000075	0.0058642	ENSG00000113140	SPARC	pre/post treatments
16.98507	1.7605036	0.3934346	4.474704	0.0000077	0.0058642	ENSG00000163735	CXCL5	pre/post treatments
53.62960	0.9521537	0.2132292	4.465400	0.0000080	0.0058697	ENSG00000130702	LAMA5	pre/post treatments
70.10139	1.5986372	0.3599056	4.441823	0.0000089	0.0062893	ENSG00000259207	ITGB3	pre/post treatments
542.07289	-0.9512056	0.2189031	-4.345328	0.0000139	0.0093261	ENSG00000163464	CXCR1	pre/post treatments
277.28727	1.4358504	0.3308845	4.339431	0.0000143	0.0093261	ENSG00000005961	ITGA2B	pre/post treatments
23.07633	1.5776817	0.3683289	4.283350	0.0000184	0.0113192	ENSG00000187800	PEAR1	pre/post treatments
70.03848	1.1200818	0.2616525	4.280799	0.0000186	0.0113192	ENSG00000269404	SPIB	pre/post treatments
196.39063	-0.9211014	0.2155759	-4.272746	0.0000193	0.0113447	ENSG00000211807	TRAV26-1	pre/post treatments
185.72847	1.0893015	0.2567862	4.242057	0.0000221	0.0125937	ENSG00000167483	NIBAN3	pre/post treatments
26.40457	1.4040611	0.3351253	4.189660	0.0000279	0.0153891	ENSG00000128438	TBC1D27P	pre/post treatments
29.54723	1.7286873	0.4149532	4.165982	0.0000310	0.0165595	ENSG00000169704	GP9	pre/post treatments
14.81421	1.2597353	0.3044415	4.137856	0.0000351	0.0181748	ENSG00000233614	DDX11L10	pre/post treatments
70.09865	1.0083057	0.2459089	4.100322	0.0000413	0.0202402	ENSG00000144645	OSBPL10	pre/post treatments
210.11370	-0.9378471	0.2287501	-4.099876	0.0000413	0.0202402	ENSG00000125355	TMEM255A	pre/post treatments
31.56092	0.6725071	0.1657311	4.057822	0.0000495	0.0230721	ENSG00000113924	HGD	pre/post treatments
108.94644	0.7577123	0.1867734	4.056853	0.0000497	0.0230721	ENSG00000211776	TRAV2	pre/post treatments
434.93407	0.8530848	0.2113974	4.035456	0.0000545	0.0246310	ENSG00000136573	BLK	pre/post treatments

Table 4: Supplementary Table 4 *(continued)*

baseMean	log2FoldChange	lfcSE	stat	pvalue	padj	ENSG.gene	Gene	dataset
118.23820	0.7903965	0.1996618	3.958677	0.0000754	0.0328101	ENSG00000187699	C2orf88	pre/post treatments
26.79648	1.4323837	0.3621076	3.955685	0.0000763	0.0328101	ENSG00000162367	TAL1	pre/post treatments
10.27013	1.3329395	0.3382627	3.940545	0.0000813	0.0340675	ENSG00000197385	ZNF860	pre/post treatments
12.04123	-1.6466976	0.4185772	-3.934035	0.0000835	0.0340675	ENSG00000169083	AR	pre/post treatments
32.58051	0.8757776	0.2228590	3.929739	0.0000850	0.0340675	ENSG00000081052	COL4A4	pre/post treatments
16.55504	1.5597662	0.3977782	3.921196	0.0000881	0.0345139	ENSG00000088726	TMEM40	pre/post treatments
69.26886	-0.5785296	0.1494378	-3.871374	0.0001082	0.0403520	ENSG00000276791	AC092117.1	pre/post treatments
15.92981	1.4946921	0.3862370	3.869883	0.0001089	0.0403520	ENSG00000170365	SMAD1	pre/post treatments
58.21459	1.4234134	0.3687541	3.860061	0.0001134	0.0403520	ENSG00000184702	SEPTIN5	pre/post treatments
25.35117	1.4854537	0.3850414	3.857907	0.0001144	0.0403520	ENSG00000173210	ABLIM3	pre/post treatments
670.17515	0.9987478	0.2588975	3.857696	0.0001145	0.0403520	ENSG00000007350	TKTL1	pre/post treatments
25.42894	-1.2501778	0.3301979	-3.786147	0.0001530	0.0528814	ENSG00000121742	GJB6	pre/post treatments
131.88399	0.3998105	0.1057746	3.779835	0.0001569	0.0531970	ENSG00000123427	EEF1AKMT3	pre/post treatments
26.49725	-0.7896811	0.2094973	-3.769409	0.0001636	0.0532799	ENSG00000167693	NXN	pre/post treatments
54.17453	1.1180680	0.2967665	3.767500	0.0001649	0.0532799	ENSG00000119866	BCL11A	pre/post treatments
42.09527	1.2568764	0.3337912	3.765457	0.0001662	0.0532799	ENSG00000049323	LTBP1	pre/post treatments
976.46331	-0.8321298	0.2218632	-3.750644	0.0001764	0.0555190	ENSG00000180871	CXCR2	pre/post treatments
16.43811	0.8475398	0.2269828	3.733938	0.0001885	0.0582956	ENSG00000279841	AC092135.3	pre/post treatments
40.49340	-1.3436010	0.3610668	-3.721198	0.0001983	0.0593385	ENSG00000165730	STOX1	pre/post treatments

Table 4: Supplementary Table 4 *(continued)*

baseMean	log2FoldChange	lfcSE	stat	pvalue	padj	ENSG.gene	Gene	dataset
169.52340	0.8067857	0.2168821	3.719927	0.0001993	0.0593385	ENSG00000124772	CPNE5	pre/post treatments
30.17458	0.6799360	0.1831494	3.712466	0.0002052	0.0593385	ENSG00000165028	NIPSNAP3B	pre/post treatments
296.89628	0.7638011	0.2057462	3.712346	0.0002053	0.0593385	ENSG00000247982	LINC00926	pre/post treatments
39.93177	-1.2554447	0.3411552	-3.679981	0.0002333	0.0662433	ENSG00000211813	TRAV34	pre/post treatments
29.40442	1.1228117	0.3054294	3.676175	0.0002368	0.0662433	ENSG00000187912	CLEC17A	pre/post treatments
16.05252	1.4708197	0.4006645	3.670951	0.0002416	0.0665555	ENSG00000171611	PTCRA	pre/post treatments
12.74257	1.4143872	0.3861864	3.662447	0.0002498	0.0671182	ENSG00000250334	LINC00989	pre/post treatments
39.26502	1.2608679	0.3444125	3.660924	0.0002513	0.0671182	ENSG00000088826	SMOX	pre/post treatments
298.19609	-0.8492064	0.2324238	-3.653697	0.0002585	0.0675544	ENSG00000125498	KIR2DL1	pre/post treatments
54.20614	1.1637965	0.3187082	3.651606	0.0002606	0.0675544	ENSG00000161911	TREML1	pre/post treatments
39.68961	0.8319217	0.2284748	3.641197	0.0002714	0.0693261	ENSG00000255354	NA	pre/post treatments
39.37267	-0.9099754	0.2506114	-3.631021	0.0002823	0.0710876	ENSG00000166444	DENND2B	pre/post treatments
147.97320	0.6752440	0.1864993	3.620625	0.0002939	0.0729640	ENSG00000241106	HLA-DOB	pre/post treatments
101.61242	1.2553529	0.3479804	3.607539	0.0003091	0.0756774	ENSG00000108846	ABCC3	pre/post treatments
165.31213	0.9306680	0.2585827	3.599112	0.0003193	0.0764351	ENSG00000226777	FAM30A	pre/post treatments
47.52429	-1.1730542	0.3260449	-3.597830	0.0003209	0.0764351	ENSG00000151640	DPYSL4	pre/post treatments
17.42185	1.1623731	0.3241011	3.586453	0.0003352	0.0787826	ENSG00000197705	KLHL14	pre/post treatments
29.60976	-1.2070289	0.3380807	-3.570239	0.0003567	0.0827206	ENSG00000166831	RBPMS2	pre/post treatments
226.23385	0.8332892	0.2340000	3.561066	0.0003694	0.0845530	ENSG00000110777	POU2AF1	pre/post treatments

Table 4: Supplementary Table 4 *(continued)*

baseMean	log2FoldChange	lfcSE	stat	pvalue	padj	ENSG.gene	Gene	dataset
90.20384	1.0820917	0.3052955	3.544408	0.0003935	0.0889251	ENSG00000132704	FCRL2	pre/post treatments
272.73786	0.6873240	0.1942848	3.537714	0.0004036	0.0900192	ENSG00000141753	IGFBP4	pre/post treatments
16.70212	1.3164380	0.3724541	3.534497	0.0004086	0.0900192	ENSG00000153162	BMP6	pre/post treatments
25.45098	1.4017798	0.3997458	3.506678	0.0004537	0.0987414	ENSG00000239961	LILRA4	pre/post treatments
828.07307	-1.3680150	0.3910685	-3.498147	0.0004685	0.0998070	ENSG00000162747	FCGR3B	pre/post treatments
107.64369	0.8885091	0.2540733	3.497058	0.0004704	0.0998070	ENSG00000074416	MGLL	pre/post treatments
586.78616	-0.8500909	0.2432916	-3.494123	0.0004756	0.0998070	ENSG00000124920	MYRF	pre/post treatments
205.14343	3.3755459	0.5644341	5.980407	0.0000000	0.0000392	ENSG00000158578	ALAS2	pretreatment sICB
91.69281	3.8096405	0.7145386	5.331609	0.0000001	0.0008565	ENSG00000004939	SLC4A1	pretreatment sICB
32.48479	4.1515907	0.8006524	5.185260	0.0000002	0.0010471	ENSG00000223609	HBD	pretreatment sICB
1749.77903	2.7847778	0.5401332	5.155724	0.0000003	0.0010471	ENSG00000188536	HBA2	pretreatment sICB
23.18379	3.3313748	0.6500221	5.125018	0.0000003	0.0010471	ENSG00000133742	CA1	pretreatment sICB
27.01643	2.6453493	0.5257404	5.031664	0.0000005	0.0014261	ENSG00000174099	MSRB3	pretreatment sICB
8012.23396	2.5655150	0.5184068	4.948845	0.0000007	0.0018767	ENSG00000244734	HBB	pretreatment sICB
678.31525	2.7558850	0.5615680	4.907482	0.0000009	0.0020292	ENSG00000206172	HBA1	pretreatment sICB
37.11543	3.1018831	0.6399799	4.846844	0.0000013	0.0024526	ENSG00000133063	CHIT1	pretreatment sICB
13.82979	1.8087792	0.3765548	4.803496	0.0000016	0.0026786	ENSG00000197744	PTMAP2	pretreatment sICB
34.84728	2.5659596	0.5357799	4.789205	0.0000017	0.0026786	ENSG00000142583	SLC2A5	pretreatment sICB
22.84216	2.6890719	0.5639748	4.768071	0.0000019	0.0027275	ENSG00000065618	COL17A1	pretreatment sICB

Table 4: Supplementary Table 4 *(continued)*

baseMean	log2FoldChange	lfcSE	stat	pvalue	padj	ENSG.gene	Gene	dataset
102.34091	3.5539583	0.7547002	4.709100	0.0000025	0.0033680	ENSG00000179869	ABCA13	pretreatment sICB
278.31167	3.1216306	0.6655162	4.690540	0.0000027	0.0034249	ENSG00000005381	MPO	pretreatment sICB
531.00443	2.8857638	0.6182718	4.667468	0.0000030	0.0035773	ENSG00000073756	PTGS2	pretreatment sICB
30.42128	3.3890085	0.7288524	4.649787	0.0000033	0.0036544	ENSG00000214548	MEG3	pretreatment sICB
23.08823	3.2340220	0.7146210	4.525506	0.0000060	0.0062367	ENSG00000250771	LOC100419170	pretreatment sICB
167.62680	1.6899951	0.3776767	4.474714	0.0000077	0.0074801	ENSG00000181409	AATK	pretreatment sICB
35.58374	2.0170134	0.4565375	4.418067	0.0000100	0.0090069	ENSG00000163053	SLC16A14	pretreatment sICB
109.87211	2.5231740	0.5718747	4.412110	0.0000102	0.0090069	ENSG00000172232	AZU1	pretreatment sICB
168.34162	1.4830092	0.3383206	4.383444	0.0000117	0.0097888	ENSG00000129757	CDKN1C	pretreatment sICB
192.45780	2.5451414	0.5866146	4.338695	0.0000143	0.0114646	ENSG00000149516	MS4A3	pretreatment sICB
120.52296	2.5646950	0.5925818	4.328002	0.0000150	0.0115121	ENSG00000134827	TCN1	pretreatment sICB
11.77149	3.0791249	0.7170182	4.294347	0.0000175	0.0125248	ENSG00000157554	ERG	pretreatment sICB
229.50537	3.1578427	0.7359371	4.290914	0.0000178	0.0125248	ENSG00000124469	CEACAM8	pretreatment sICB
15.22109	3.5365027	0.8296756	4.262513	0.0000202	0.0136811	ENSG00000184292	TACSTD2	pretreatment sICB
25.06177	1.5176950	0.3626735	4.184742	0.0000285	0.0182476	ENSG00000066056	TIE1	pretreatment sICB
413.69120	1.2193999	0.2916594	4.180904	0.0000290	0.0182476	ENSG00000247982	LINC00926	pretreatment sICB
53.59454	1.0712674	0.2590078	4.136043	0.0000353	0.0214408	ENSG00000020181	ADGRA2	pretreatment sICB
38.66590	2.1245733	0.5172289	4.107608	0.0000400	0.0234497	ENSG00000258227	CLEC5A	pretreatment sICB
47.75639	-2.5429374	0.6221077	-4.087616	0.0000436	0.0243460	ENSG00000281181	FP236383.5	pretreatment sICB

Table 4: Supplementary Table 4 *(continued)*

baseMean	log2FoldChange	lfcSE	stat	pvalue	padj	ENSG.gene	Gene	dataset
114.46192	3.2107539	0.7861848	4.083968	0.0000443	0.0243460	ENSG00000086548	CEACAM6	pretreatment sICB
349.31291	2.6751066	0.6699213	3.993165	0.0000652	0.0347658	ENSG00000101425	BPI	pretreatment sICB
51.25708	1.7511246	0.4430511	3.952422	0.0000774	0.0400406	ENSG00000119862	LGALSL	pretreatment sICB
453.71752	2.6788737	0.6805395	3.936397	0.0000827	0.0415862	ENSG00000148346	LCN2	pretreatment sICB
36.27608	3.0546003	0.7884793	3.874040	0.0001070	0.0523246	ENSG00000173391	OLR1	pretreatment sICB
49.58631	1.4575312	0.3772136	3.863942	0.0001116	0.0523909	ENSG00000106537	TSPAN13	pretreatment sICB
74.71861	2.3823618	0.6174205	3.858572	0.0001141	0.0523909	ENSG00000170956	CEACAM3	pretreatment sICB
667.73141	-1.4786097	0.3836369	-3.854190	0.0001161	0.0523909	ENSG00000124920	MYRF	pretreatment sICB
344.97268	2.6623716	0.6921062	3.846767	0.0001197	0.0526531	ENSG00000196549	MME	pretreatment sICB
100.35696	3.3589698	0.8822193	3.807409	0.0001404	0.0602720	ENSG00000197561	ELANE	pretreatment sICB
2665.28973	2.4949056	0.6568374	3.798361	0.0001457	0.0610264	ENSG00000012223	LTF	pretreatment sICB
12.04944	2.4652565	0.6509107	3.787396	0.0001522	0.0622993	ENSG00000096696	DSP	pretreatment sICB
266.44877	1.0083090	0.2671821	3.773864	0.0001607	0.0637527	ENSG00000104043	ATP8B4	pretreatment sICB
65.59870	2.3397335	0.6205646	3.770330	0.0001630	0.0637527	ENSG00000233392	UICLM	pretreatment sICB
395.11128	1.3894419	0.3702477	3.752737	0.0001749	0.0669125	ENSG00000012124	CD22	pretreatment sICB
84.36126	1.5149343	0.4060277	3.731111	0.0001906	0.0713754	ENSG00000241351	IGKV3-11	pretreatment sICB
115.75168	1.5612843	0.4198158	3.718974	0.0002000	0.0733331	ENSG00000104921	FCER2	pretreatment sICB
47.05493	1.6227173	0.4378801	3.705848	0.0002107	0.0756616	ENSG00000171798	KNDC1	pretreatment sICB
70.62204	1.2216238	0.3301863	3.699802	0.0002158	0.0759374	ENSG00000130702	LAMA5	pretreatment sICB

Table 4: Supplementary Table 4 *(continued)*

baseMean	log2FoldChange	lfcSE	stat	pvalue	padj	ENSG.gene	Gene	dataset
12.15918	-1.7367178	0.4707079	-3.689587	0.0002246	0.0768727	ENSG00000132938	MTUS2	pretreatment sICB
1205.52978	0.7136698	0.1935785	3.686721	0.0002272	0.0768727	ENSG00000148180	GSN	pretreatment sICB
143.46026	2.4941959	0.6784458	3.676337	0.0002366	0.0785578	ENSG00000186529	CYP4F3	pretreatment sICB
153.84232	2.4360611	0.6645818	3.665555	0.0002468	0.0804257	ENSG00000138772	ANXA3	pretreatment sICB
263.48132	1.9408778	0.5307089	3.657142	0.0002550	0.0815998	ENSG00000272398	CD24	pretreatment sICB
107.86951	0.6469718	0.1774065	3.646834	0.0002655	0.0834260	ENSG00000139531	SUOX	pretreatment sICB
10.56919	-1.9290234	0.5297804	-3.641175	0.0002714	0.0837852	ENSG00000266717	AC134407.1	pretreatment sICB
14.13618	1.7896640	0.4929742	3.630340	0.0002830	0.0851311	ENSG00000168916	ZNF608	pretreatment sICB
27.86464	2.4364376	0.6725898	3.622472	0.0002918	0.0851311	ENSG00000226321	CROCC2	pretreatment sICB
49.31656	1.8007942	0.4971566	3.622187	0.0002921	0.0851311	ENSG00000148926	ADM	pretreatment sICB
146.64252	3.0338429	0.8384187	3.618530	0.0002963	0.0851311	ENSG00000164821	DEFA4	pretreatment sICB
344.11329	-0.9493855	0.2625986	-3.615348	0.0002999	0.0851311	ENSG00000137970	RPL7P9	pretreatment sICB
373.16400	2.5294062	0.7008145	3.609238	0.0003071	0.0857778	ENSG00000118113	MMP8	pretreatment sICB
86.56198	-1.5842476	0.4401883	-3.599023	0.0003194	0.0878242	ENSG00000061337	LZTS1	pretreatment sICB
51.13877	1.5622391	0.4358543	3.584315	0.0003380	0.0914947	ENSG00000153208	MERTK	pretreatment sICB
141.89167	1.6929635	0.4748074	3.565580	0.0003631	0.0966872	ENSG00000108846	ABCC3	pretreatment sICB
287.12594	-1.8120601	0.5092074	-3.558589	0.0003729	0.0966872	ENSG00000114948	ADAM23	pretreatment sICB
31.97469	2.2826885	0.6415573	3.558043	0.0003736	0.0966872	ENSG00000244575	IGKV1-27	pretreatment sICB
18.27375	2.7576490	0.7764363	3.551674	0.0003828	0.0970982	ENSG00000163739	CXCL1	pretreatment sICB

Table 4: Supplementary Table 4 *(continued)*

baseMean	log2FoldChange	lfcSE	stat	pvalue	padj	ENSG.gene	Gene	dataset
89.17322	0.7825299	0.2206607	3.546303	0.0003907	0.0970982	ENSG00000128311	TST	pretreatment sICB
30.12218	-0.6028879	0.1702003	-3.542225	0.0003968	0.0970982	ENSG00000232053	AC078845.1	pretreatment sICB
189.58411	2.1764384	0.6144870	3.541879	0.0003973	0.0970982	ENSG00000123689	G0S2	pretreatment sICB

Table 5: GOBP pathway analysis

	name	nAnno	nOverlap	fc	zscore	pvalue	adjp	or	CIl	CIu	dataset
GO:0006955	immune response	282	18	4.99	7.72	0.0e+00	1.0e-07	5.87	3.320	9.83	pre/post treatment
GO:0030198	extracellular matrix organization	110	11	7.81	8.18	0.0e+00	2.0e-07	9.19	4.340	17.70	pre/post treatment
GO:0002576	platelet degranulation	94	9	7.48	7.18	3.0e-07	3.0e-06	8.64	3.740	17.70	pre/post treatment
GO:0007596	blood coagulation	109	9	6.45	6.51	1.2e-06	9.0e-06	7.34	3.190	14.90	pre/post treatment
GO:0030168	platelet activation	87	8	7.19	6.59	1.6e-06	9.1e-06	8.21	3.360	17.40	pre/post treatment
GO:0006954	inflammatory response	254	13	4.00	5.50	4.5e-06	2.2e-05	4.47	2.290	8.06	pre/post treatment
GO:0070098	chemokine-mediated signaling pathway	36	5	10.90	6.74	5.6e-06	2.3e-05	12.80	3.840	34.00	pre/post treatment
GO:0007229	integrin-mediated signaling pathway	68	6	6.89	5.55	2.5e-05	8.9e-05	7.74	2.690	18.20	pre/post treatment
GO:0007166	cell surface receptor signaling pathway	165	9	4.26	4.81	5.0e-05	1.6e-04	4.68	2.060	9.38	pre/post treatment
GO:0006935	chemotaxis	77	5	5.07	4.08	4.4e-04	1.2e-03	5.51	1.710	13.80	pre/post treatment
GO:0007186	G-protein coupled receptor signaling pathway	216	9	3.26	3.81	4.5e-04	1.2e-03	3.51	1.550	6.99	pre/post treatment
GO:0042127	regulation of cell proliferation	132	6	3.55	3.36	1.5e-03	3.7e-03	3.79	1.340	8.69	pre/post treatment
GO:0007155	cell adhesion	256	9	2.75	3.22	1.7e-03	3.7e-03	2.93	1.300	5.82	pre/post treatment
GO:0010628	positive regulation of gene expression	184	7	2.97	3.07	2.5e-03	5.2e-03	3.15	1.230	6.81	pre/post treatment
GO:0002250	adaptive immune response	113	5	3.46	2.99	3.3e-03	6.2e-03	3.66	1.150	9.02	pre/post treatment
GO:0032496	response to lipopolysaccharide	114	5	3.43	2.97	3.4e-03	6.2e-03	3.63	1.140	8.93	pre/post treatment
GO:0050776	regulation of immune response	135	5	2.89	2.52	7.7e-03	1.3e-02	3.04	0.955	7.44	pre/post treatment
GO:0007165	signal transduction	727	15	1.61	1.94	2.3e-02	3.8e-02	1.70	0.918	2.93	pre/post treatment
GO:0045893	positive regulation of transcription, DNA-templated	388	9	1.81	1.85	2.7e-02	4.2e-02	1.89	0.838	3.73	pre/post treatment
GO:0008285	negative regulation of cell proliferation	285	7	1.92	1.79	3.0e-02	4.4e-02	1.99	0.778	4.27	pre/post treatment

Table 5: Supplementary Table 5 *(continued)*

	name	nAnno	nOverlap	fc	zscore	pvalue	adjp	or	CIl	CIu	dataset
GO:0043312	neutrophil degranulation	450	108	3.28	13.90	0.0e+00	0.0e+00	4.46	3.510	5.63	pretreatment sICB
GO:00069541	inflammatory response	254	61	3.29	10.30	0.0e+00	0.0e+00	4.25	3.100	5.75	pretreatment sICB
GO:0042742	defense response to bacterium	75	30	5.47	10.90	0.0e+00	0.0e+00	8.73	5.280	14.30	pretreatment sICB
GO:00069551	immune response	287	60	2.86	8.96	0.0e+00	0.0e+00	3.54	2.590	4.77	pretreatment sICB
GO:0019730	antimicrobial humoral response	25	16	8.76	10.90	0.0e+00	0.0e+00	23.00	9.520	59.10	pretreatment sICB
GO:00324961	response to lipopolysaccharide	116	34	4.01	9.15	0.0e+00	0.0e+00	5.44	3.510	8.27	pretreatment sICB
GO:0045087	innate immune response	305	56	2.51	7.52	0.0e+00	0.0e+00	2.99	2.170	4.05	pretreatment sICB
GO:0050900	leukocyte migration	146	35	3.28	7.79	0.0e+00	0.0e+00	4.13	2.720	6.14	pretreatment sICB
GO:0050853	B cell receptor signaling pathway	56	20	4.89	8.19	0.0e+00	0.0e+00	7.19	3.930	12.80	pretreatment sICB
GO:0006910	phagocytosis, recognition	30	14	6.38	8.29	0.0e+00	0.0e+00	11.30	5.070	24.70	pretreatment sICB
GO:0006911	phagocytosis, engulfment	36	15	5.70	7.93	0.0e+00	0.0e+00	9.20	4.400	18.80	pretreatment sICB
GO:0006958	complement activation, classical pathway	78	22	3.86	7.11	0.0e+00	1.0e-07	5.09	2.940	8.52	pretreatment sICB
GO:00071861	G-protein coupled receptor signaling pathway	218	40	2.51	6.32	0.0e+00	4.0e-07	2.94	2.020	4.20	pretreatment sICB
GO:0050871	positive regulation of B cell activation	29	12	5.66	7.06	0.0e+00	8.0e-07	9.07	3.940	20.20	pretreatment sICB
GO:0002224	toll-like receptor signaling pathway	35	13	5.08	6.79	1.0e-07	1.3e-06	7.59	3.500	15.80	pretreatment sICB
GO:00022501	adaptive immune response	118	26	3.01	6.18	1.0e-07	1.4e-06	3.67	2.260	5.76	pretreatment sICB
GO:00071551	cell adhesion	259	43	2.27	5.81	1.0e-07	1.9e-06	2.61	1.820	3.66	pretreatment sICB
GO:0006956	complement activation	55	16	3.98	6.22	2.0e-07	3.4e-06	5.28	2.740	9.73	pretreatment sICB
GO:0045766	positive regulation of angiogenesis	74	19	3.51	6.09	2.0e-07	3.4e-06	4.46	2.490	7.68	pretreatment sICB
GO:0001774	microglial cell activation	12	7	7.98	6.79	3.0e-07	4.7e-06	17.90	4.870	71.60	pretreatment sICB
GO:00075961	blood coagulation	109	23	2.89	5.56	8.0e-07	1.2e-05	3.46	2.070	5.57	pretreatment sICB

Table 5: Supplementary Table 5 *(continued)*

	name	nAnno	nOverlap	fc	zscore	pvalue	adjp	or	CIl	CIu	dataset
GO:0050729	positive regulation of inflammatory response	38	12	4.32	5.76	1.5e-06	2.1e-05	5.92	2.710	12.20	pretreatment sICB
GO:0006898	receptor-mediated endocytosis	144	27	2.57	5.31	1.5e-06	2.1e-05	2.99	1.880	4.61	pretreatment sICB
GO:00507761	regulation of immune response	137	26	2.60	5.28	1.7e-06	2.3e-05	3.03	1.890	4.72	pretreatment sICB
GO:0019731	antibacterial humoral response	15	7	6.38	5.86	3.2e-06	3.9e-05	11.20	3.440	35.40	pretreatment sICB
GO:00025761	platelet degranulation	94	20	2.91	5.22	3.1e-06	3.9e-05	3.49	2.000	5.81	pretreatment sICB
GO:0060326	cell chemotaxis	31	10	4.41	5.34	6.5e-06	7.4e-05	6.10	2.560	13.60	pretreatment sICB
GO:00072291	integrin-mediated signaling pathway	69	16	3.17	5.08	6.6e-06	7.4e-05	3.88	2.060	6.94	pretreatment sICB
GO:0030593	neutrophil chemotaxis	37	11	4.07	5.25	7.2e-06	7.9e-05	5.42	2.410	11.40	pretreatment sICB
GO:0050829	defense response to Gram-negative bacterium	22	8	4.97	5.24	1.2e-05	1.2e-04	7.30	2.650	18.70	pretreatment sICB
